# Long-Term Clinical Outcomes of a Remote Digital Musculoskeletal Program: An Ad Hoc Analysis from a Longitudinal Study with a Non-Participant Comparison Group

**DOI:** 10.3390/healthcare10122349

**Published:** 2022-11-23

**Authors:** Anabela C. Areias, Fabíola Costa, Dora Janela, Maria Molinos, Robert G. Moulder, Jorge Lains, Justin K. Scheer, Virgílio Bento, Vijay Yanamadala, Fernando Dias Correia

**Affiliations:** 1 Sword Health Inc., Draper, UT 84043, USA; 2 Institute for Cognitive Science, University of Colorado Boulder, Boulder, CO 80309, USA; 3 Rovisco Pais Medical and Rehabilitation Centre, 3064-908 Tocha, Portugal; 4 Faculty of Medicine, Coimbra University, 3004-504 Coimbra, Portugal; 5 Department of Neurological Surgery, University of California, San Francisco, CA 94143, USA; 6 Department of Surgery, Frank H. Netter School of Medicine, Quinnipiac University, Hamden, CT 06473, USA; 7 Department of Neurosurgery, Hartford Healthcare Medical Group, Westport, CT 06103, USA; 8 Neurology Department, Centro Hospitalar e Universitário do Porto, 4099-001 Porto, Portugal

**Keywords:** chronic musculoskeletal pain, physical therapy, telerehabilitation, functionality, eHealth, follow-up

## Abstract

Digital solutions have emerged as an alternative to conventional physiotherapy, particularly for chronic musculoskeletal pain (CMP) management; however, its long-term effects remain largely unexplored. This study focuses on patients reporting CMP, assessing 1-year clinical outcomes after a multimodal digital care program (DCP) versus non-participants, who enrolled in the program but never engaged in a single exercise session or partook of the educational content made available to them. In this longitudinal study ad-hoc analysis, pain and functionality outcomes at 1-year reassessment were studied, focusing on the odds of reaching minimal clinically important difference (MCID) and, overall average reduction in both outcomes. Healthcare utilization was additionally studied within the same period. From 867 individuals allocated to the study, 460 completed the 1-year reassessment (intervention group: 310; comparison group: 150). The intervention group presented sustained and greater pain reduction until 1-year reassessment than the comparison group, reflecting greater odds ratio of achieving the MCID both in pain and functionality (1.90 95% CI: 1.27–2.86, *p* = 0.002 and 2.02 95% CI: 1.31–3.12, *p* = 0.001, respectively). A lower healthcare utilization during the 1-year follow-up was observed in the intervention group than in the comparison group. This study suggests that a digital CMP program may have a lasting impact on improved pain and functionality in patients with CMP.

## 1. Introduction

Musculoskeletal (MSK) disorders are the main cause of disability worldwide, being collectively responsible for nearly 150 million years lived with disability (YLD), a figure that increased by 59% since 1990 [1].

The burden of MSK disorders is mainly driven by chronic musculoskeletal pain (CMP), i.e., pain that lasts beyond 3 months [2,3]. The anatomical regions most frequently affected by CMP are back, shoulder and knee [4].

CMP is associated with tremendous economic impact, with total costs estimated at approximately $560–635 billions in the US, exceeding those for heart diseases and cancer [5]. Patients suffering from CMP have diminished quality of life, impaired job performance (often resulting in loss of employment, early retirement, and disability), and compromised ability to perform daily activities [6]. Furthermore, CMP is often associated with poor mental health in a bidirectional feedback loop [7]. Depression and/or anxiety may aggravate pain, which, in turn, can compromise pain management by potentiating catastrophizing and fear-avoidance beliefs [8,9,10].

Non-pharmacological interventions combining exercise, education and behavior modification strategies are widely recommended as a first approach to CMP [11,12,13,14]. These approaches have been shown to be effective in reducing pain and improving functionality, quality of life and mental health [15,16,17,18,19].

Access barriers to rehabilitation, such as travel constraints, costs and provider availability, are major contributors to this problem [20,21,22], all of which got worse during the COVID pandemic [23,24]. Further compounding this problem is poor adherence to physical therapy, contributing to prolonged recovery time, medical complications, and increased costs of care [25,26,27].

Telerehabilitation has emerged as a great alternative for MSK pain management, offering a cost-effective and resource-efficient option [26,28]. Furthermore, digital interventions allow easy delivery of multimodal approaches, highly recommended for CMP [6,29]. Telerehabilitation for CMP has shown comparable results to in-person physical therapy [30,31,32,33,34,35], while reducing costs [36,37,38]. Patient participation [39] and adherence [40] may also be maximized through these approaches.

Although there is growing evidence for the impact of digital health interventions on MSK outcomes, most trials focus on short or medium-term outcomes [41,42,43,44,45,46,47,48,49,50,51], leaving a gap in the long-term impact of digital interventions on CMP [31,32,33,34,35].

We have developed a multimodal digital care program (DCP) combining exercise education and cognitive behavioral therapy (CBT), which has been previously validated in chronic and acute MSK conditions [52,53,54,55,56,57]. Compared to in-person approaches, this DCP has demonstrated at least similar impact on pain and functionality in both the short and long term in the context of post-surgical rehabilitation [56,57,58,59].

In the present study, we aim to assess long-term clinical outcomes of patients reporting CMP who enrolled in this DCP compared to those who enrolled in the program but never engaged in a single exercise session or partook of the educational content made available to them. We hypothesized that participants who enrolled in the multimodal DCP would report better clinical outcomes at 1-year following the intervention than participants from the comparison group.

## 2. Materials and Methods

### 2.1. Study Design

This is an ad-hoc analysis of a decentralized, longitudinal prospective study, which was registered on ClinicalTrials.gov (NCT04092946) on 17 September 2019 and approved by the New England Institutional Review Board on 18 June 2020. This study was focused on the assessment of clinical and engagement-related outcomes following a remote multimodal DCP in patients with CMP. To evaluate long-term outcomes, participants that started their program one year prior were selected to the study. Therefore, the home-based DCP was delivered between 7 January 2021 and 13 May 2021, with non-participant patients enrolling within the same time period.

### 2.2. Participants

Adults (>18 years of age) beneficiaries of a health plan reporting chronic MSK pain (CMP) (≥12 weeks) in any of the following body areas: ankle, elbow, hip, knee, low back, neck, shoulder, and wrist/hand, were invited to apply for Sword Health’s DCP (Draper, Utah, USA) through a dedicated website. Individuals were excluded if: (1) they had a health condition (e.g., cardiac, respiratory) incompatible with at least 20 min of light to moderate exercise; (2) were undergoing treatment for active cancer; or (3) reported rapidly progressive loss of strength and/or numbness in the arms/legs, or an unexplained change in bowel or urinary function in the previous 2 weeks. All participants provided written informed consent. All participants who started the program a year prior were encouraged to submit 1-year follow-up reassessment surveys, through an email campaign offering $20 gift cards, with friendly reminders in the following 2-weeks. Baseline, 12-weeks and 1-year data was collected via email. The intervention group consisted of participants who engaged in the DCP program. Early discharge was possible depending on the physical therapist’s (PT) clinical judgment. The comparison group corresponded to participants who registered for the program but did not start the intervention (never engaged in a single exercise session or partook of the educational content made available to them).

### 2.3. Intervention

The DCP, previously described elsewhere [53,54,60], consisted of an 8 or 12-week program comprising exercise, education and cognitive behavioral therapy (CBT). Upon enrollment each participant was assigned to a PT. A total of 56 PT were involved in the study. Each patient received a Digital Therapist (DT), an FDA-listed class II medical device consisting of a tablet with a pre-installed app (with instructional videos), that uses motion tracking technology to digitize motion and to provide real-time biofeedback during exercise. It was recommended that participants perform at least 3 exercise sessions per week. The DT connects with a cloud-based platform, which enables the PT to asynchronously monitor progress and adjust the protocol as per clinical judgment. The exercise program followed pre-determined phases, which were adapted by the PT to each participant’s particular progress. Exercises included gradual exposure to movements through mobility/stretching, strength, and balance. Phase progress would include an increase of: (i) number of repetitions and sets, (ii) external load, (iii) range of motion, and (iv) multi-articular and multi-directional exercises. The education and CBT components were developed according to current clinical guidelines and research [12,15,61,62,63], focusing on topics related to anatomy and physiology, evidence-based treatments, prognosis of symptoms, importance of staying active and fear-avoidance. The CBT program, based on third generation techniques (mindfulness, acceptance and commitment therapy and empathy-focused therapy) consisted of self-guided interactive modules specifically designed to address pain catastrophizing, active coping methods, and fear-avoidance. Bi-directional communication was ensured through a built-in secure chat within a smartphone app and video calls. The education content provided was condition-specific, while the CBT was generally MSK pain-oriented.

### 2.4. Outcome Measures

The following outcomes were collected at baseline, 12-weeks, and 1-year for the intervention group, and at baseline and 1-year for the comparison group:

(i) Pain using the Numerical Pain Rating Scale (NPRS), through the question: “Please rate your average pain over the last 7 days” from 0 (no pain at all) to 10 (worst pain imaginable)” [64]. Following the IMMPACT recommendations [65], a 30% pain improvement was considered as a minimal clinically important difference (MCID).

(ii) Functionality, using one of the following questionnaires, corresponded to each participant’s self-reported condition: Quick Disabilities of the Arm, Shoulder and Hand questionnaire (QuickDASH) [66], the shortened versions of Hip Disability and Osteoarthritis Outcome Score (HOOS-PS) [67] and Knee Injury and Osteoarthritis Outcome Score—Physical Function (KOOS-PS) [67], Oswestry Disability Index (ODI) [68], Neck Disability Index (NDI) [69], and Quick Disabilities for Foot and Ankle Ability Measure (Quick-FAAM) [70]. Reaching a MCID in functional improvement was given by a binary variable (Yes/No) defined by meeting an MCID of 30% for QuickDASH [71]; 10-point difference for HOOS-PS [72]; a 4% change for KOOS-PS [73]; a 30% change for ODI [68]; a 5.5-point change for NDI [74]; and a 6.5% change for Quick-FAAM [70].

(iii) Health care utilization through the question “Specify additional care if you seek additional care for your condition.” (Conservative care: face-to-face physical therapy and chiropractor/osteopath; Invasive or emergency care: injections, emergency room and surgery).

### 2.5. Safety and Adverse Events

Patients were advised to report any adverse events [75] to the dedicated PT through the available communication channels for further assessment. Additionally, pain and fatigue levels during the exercise sessions (assessed by Numeric Rating Scale, range 0–10) were collected at the end of each session.

### 2.6. Data Availability

All relevant data is provided in the article or as supplementary material. The protocol, de-identified data and analysis codes may be provided upon request to the corresponding author.

### 2.7. Sample Size

A power analysis by simulation was conducted to estimate the proper sample size necessary to detect significant changes over time in pain above and beyond the minimal clinically important difference (see outcome section) after 12-weeks. Simulated data was generated using the lavaan package within the R statistical computing environment. Sample sizes in this simulation ranged from 25 to 300 participants. For each sample size, 10,000 simulations were conducted to assess statistical power at the *p* < 0.050 level of significance. Each simulated data set was obtained with parameters from previous studies of longitudinal change in treatment outcomes [52,53,54,55]. Additionally, a 30% attrition rate was assumed across the simulated data set. The results of this analysis showed that an initial sample of 130 participants in each group would be sufficient to achieve at least 80% statistical power to find a significant effect, greater than or equal to the MCID of 30% for pain [65].

### 2.8. Statistical Analyses

A descriptive analysis of the study population demographics (age, body mass index (BMI), gender and employment status) and clinical data at baseline (pain, functionality, anxiety by Generalized Anxiety Disorder 7-item questionnaire (GAD-7, range 0–21 [76]), and depression by Patient Health 9-item scale (PHQ-9, range 0–27) [77]) was performed. BMI categories were defined following Weir et al. [78]. Statistical analysis between intervention and comparison groups was performed using independent samples t-test or Mann–Whitney U test for quantitative variables, or Chi-squared test or one-sided two-proportion Z-test for qualitative variables.

To ensure comparability between groups, inverse probability weighting (IPW) was applied for controlling for confounding effects of differing baseline characteristics, as these reduce the ability of researchers to derive causal inference from clinical studies [79]. IPW differentially weights each participant’s data based upon their baseline characteristics, while keeping all data, being one of the methods recommended by ROBINS-I tool to address bias due to confounding [80]. These weights are then included during data analysis to strengthen causal inference. IPW was performed on participants in both the intervention and comparison groups based upon baseline characteristics of age, BMI, gender, pain level, depression score, and anxiety score. IPW R package was used in order to estimate all weights [81]. These weights were then used in the estimation of logistic regression models predicting if participants met MCID status based upon the study group.

To address the potential bias brought by missing data, the multiple imputation of chained equations (MICE) algorithm was applied to enable data analysis for the entire cohort at 1 year follow-up [82]. Data imputation via the MICE algorithm has been shown to be a statistically reliable means of recovering information from missing data, thereby reducing uncertainty and bias of model parameters [83]. By coupling missing data imputation via MICE and IPW, the statistical models can give more robust and reliable estimates of treatment effects while controlling for confounding. Odds ratios (OR) were calculated to compare the likelihood that an outcome will occur given a particular exposure—intervention, compared to the odds of the outcome occurring in the absence of that exposure.

All statistical analyses were conducted using commercially available software (SPSS v22, IBM, Armonk, NY, USA) or coded using R (version 1.4.1717, R Foundation for Statistical Computing). The level of significance was set at *p* < 0.05 for all tests.

## 3. Results

### 3.1. Participants

**Figure 1:**
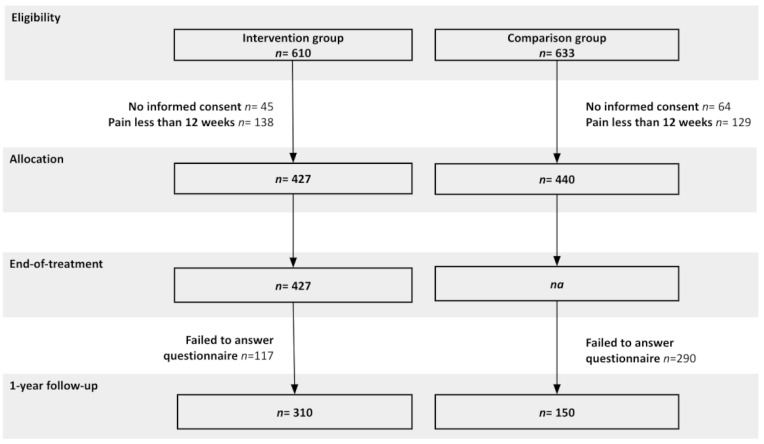
Study flow chart.

A total of 867 individuals were allocated to the study, of which 460 individuals delivered 1-year follow-up assessment, 310 belonged to the intervention group (IG) and 150 to the comparison group (CG). The study flowchart is presented in Figure 1. During the intervention, no serious adverse events [75] were reported. Patients from the intervention group performed on average 3.33 (SD 0.98) sessions per week, including 22% who performed below the 3 sessions.

### 3.2. Baseline Characteristics

Table 1 shows the demographic characteristics of the study sample. Mean age of the total cohort was 49.2 years (SD 11.4) with the majority being women (53.4%), middle age (40–60 years: 55.4%), overweight or with obesity (33.6% and 41.5%, respectively), and employed (96.3%). The most prevalent MSK condition was low back pain, which was reported by 36.6% of participants, followed by shoulder (16.0%) and knee (15.6%) conditions.

Comparing the intervention to the non-participant group, the latter was significantly younger (CG: 46.5 (SD 11.1) vs. IG: 50.5 (SD 11.3), *p* < 0.001) and presented a significantly higher proportion of patients with obesity grade III (CG: 20.7% vs. IG: 7.7%, *p* < 0.001) and patients employed part-time (CG: 78% vs. IG: 43.5%, *p* < 0.001). No significant differences in the anatomical pain region were found between both groups. Additionally, the comparison group presented higher levels of pain (CG: 4.9, 95% CI 4.6–5.2 vs. IG: 4.4, 95% CI 4.1–4.6, *p* = 0.009), depression (PHQ-9 ≥ 5: 8.04, 95% CI 7.27–8.81 vs. 10.1, 95% CI 8.59–11.63, *p* = 0.008) and anxiety (GAD-7 ≥ 5: 8.18, 95% CI 7.36–9.01 vs. 9.79, 95% CI 8.57–11.01, *p* = 0.028).

### 3.3. Clinical Outcomes

The intervention group had a pain change from 4.40 (95% CI 4.19–4.61) at baseline to 2.19 (95% CI 1.99–2.39) at the end of the 12-week program, which did not clinically differ from the obtained data for 1-year outcomes.

A significant pain decrease of −2.38 points (95% CI 1.94–2.82, *p* < 0.001) was observed in the intervention group at 1-year follow-up when considering the completers cohort, and of −2.43 points (95% CI 2.12–2.74, *p* = 0.008) when considering the entire cohort (Table 2).

Greater pain reduction was observed in the intervention group when compared to the comparison group (CC), who reported a mean change of −1.79 (95% CI 1.43–2.14 points, *p* < 0.001) in the completers cohort and of −1.78 (95% CI 1.57–2.00 points, *p* < 0.001) in the entire cohort.

Considering the recommended MCID of 30% for pain [65], a 71.6% response rate was found for the intervention group and 57.3% for the comparison group. Within completers, this translated into an odds ratio (OR) of being a responder for pain of 1.90, 95% CI 1.27–2.86, *p* = 0.002 for the intervention group when compared to the comparison group (OR 1.86 (95% CI 1.40–2.47, *p* < 0.001) for the entire cohort), Table 3. When analyzing the effect of each covariate in the odds for being a responder, only pain at baseline had a significant effect in the result (OR: 1.12, 95% CI 1.00–1.26, *p* = 0.045 Supplementary Table S1).

Regarding functionality, a 47.4% response rate for reaching functional MCID was observed for the intervention group versus 36.9% in the comparison group, respectively. Among the completers, a significant OR of 2.02 (95% CI 1.31–3.12, *p* = 0.001) for being a responder was obtained favoring the intervention group (OR 2.25 (95% CI 1.72–2.96, *p* < 0.001), entire cohort), Table 3. None of the covariates influenced the likelihood of being a responder for functionality, in the completers cohort. When analyzing the entire cohort, low OR was observed for both body mass index (BMI) (OR of 0.97 95% CI 0.95–1.00, *p* = 0.037) and invasive care (OR of 0.36 95% CI 0.16–0.80, *p* = 0.013), Supplementary Table S1. These results suggest that patients with higher BMI or those seeking invasive and emergency care had difficulty reaching MCID for functionality.

When analyzing conservative care utilization during the year, we found that the intervention group had a lower number of consultations with physical therapists and osteopaths/chiropractors than the comparison group (CG: 21.3% vs. IG: 14.8%, *p* = 0.041). A similar tendency was observed for invasive and emergency care, corresponding to usage of injections, emergency rooms and surgery, where the intervention group reported a utilization of 6.1%, versus 10.0% on the comparison group (*p* = 0.068).

## 4. Discussion

### 4.1. Main Findings

A significant pain reduction was observed in the intervention group after the digital care program (DCP), which remained stable until the 1-year reassessment, and was higher than the pain reduction observed in the comparison group. The response rate for pain was superior in the intervention group (71.6% versus 57.3%) to that which was observed in the comparison group. Similarly, the response rate for functionality was higher in the intervention group (47.4%) than in the comparison group (36.7%) at 1-year. These results, translated in a significant odds ratio (OR) for being a responder both for pain and functionality, favor the intervention group. Additionally, the intervention group reported lower conversative care utilization during the 1-year follow-up.

### 4.2. Comparison with Literature

Chronic MSK pain (CMP) affects one in every five people, representing a clinical and social problem worldwide [84]. Herein, the most common reported affected areas by both intervention and comparison groups were low back, followed by shoulder and knee, in line with the prevalence of MSK conditions found in the US general population [4]. In addition, the cohort follows a demographic pattern (mainly women, and patients who are overweight or obese) that has been previously associated to a higher likelihood of developing CMP [85,86,87,88], with a similar trend in both groups.

In the present study, pain levels reported by both groups at baseline (4.6–4.8 points) were within the range of those described in the literature for CMP both in telerehabilitation (5.8–4.5 points) [31,32,33,34,52,53,89] and in conventional physical therapy (4.6–5.8 points) studies [90,91,92]. When analyzing the impact of telerehabilitation interventions on pain reduction at 1-year, the results varied, possibly depending on a number of factors, including baseline pain severity, type of pain management strategy, length of the intervention and particularly the affected joint (e.g., several anatomic regions (50.9%) [31], low back (44.8%) [93], knee (43.9%) [32], hip (34.5%) [32], or hip and knee (17.2%) [94]). In the present study, a 2.43-points change was observed, with the comparison group reporting significantly lower improvement. Accordingly, higher response rates were observed in the intervention group (71.6% versus 57.3%) than in the comparison group. Even though we accounted for baseline differences using inverse probability weighting, we found that the likelihood of being a responder for pain at 1-year was influenced by initial pain levels, which is in accordance with previous literature [95]. Similarly, the comparison group presented higher BMI levels at baseline, which did not impact the likelihood of being a responder, despite high BMI being a known factor for poor prognosis of MSK pain management [96,97]. Direct comparison of response rates with other studies is difficult owing to different pain assessment tools and minimal clinical important change applied thresholds. Nevertheless, these results are consistent with those reported in studies evaluating the effect of digital programs on CMP in several anatomic regions [31] or in a single affected area (low back, knee and hip) [32,93,94]. For example, Kent et al. conducted a cluster pilot randomized controlled trial (RCT) to assess the effect of a multidisciplinary digital intervention (consisting of exercise, education and biofeedback) in a mixed acuity cohort (80% chronic) of patients with low back pain, reporting a response rate of 68% at 12 months in comparison to 21% in the standard care group [93]. Wang et al., in a similar intervention for chronic MSK for several anatomic regions reported a response rate of 72.2% in the intervention group, versus 56.2% in a control group composed of participants who enrolled but did not engage in any session [31]. In another longitudinal observational study, focusing on assessing the long-term outcomes (48-weeks) of a digital self-management program (composed of exercise and educational components) for hip and knee osteoarthritis, a response rate of 72% was reported [32], considering a lower minimal clinically important change of 20% for pain.

Functional long-term outcomes reported across the literature diverge, following either in-person or digital physiotherapy. In a systematic review assessing non-invasive non-pharmacological treatment, such as exercise for chronic pain, no long-term functional improvement for chronic low back pain and osteoarthritis pain was reported [98]. However, for chronic neck pain, exercise seemed to have a short- and long-term effect in improved function [98]. On the other hand, telerehabilitation studies failed to report evidence of sustained functional improvement [31,89]. In the present study, alongside pain reduction, significant long-term functional improvements were observed in the intervention group (47.4% response rate vs. 36.9% in the comparison group), as suggested by a significant OR both in completers and the entire cohort.

Significantly fewer participants sought physical therapists and osteopaths/chiropractors than non-participants (IG: 14.8% vs. CG: 21.3%, *p* = 0.041), with a similar trend regarding invasive and emergency care (injections, emergency rooms and surgery), where the intervention group reported a utilization of 6.1%, versus 10.0% of the comparison group *(p* = 0.068). One possible explanation might be that the improvements in pain and functionality experienced by patients in the intervention group may have prevented the need for other healthcare services. That is, this digital MSK program may have acted as a substitute for usual in-person care, particularly because this program was delivered during the COVID-19 pandemic. It may be reasonable to assume that the percent of additional healthcare usage was likely lower than usual in both groups, although we cannot conclude if the pandemic equally influenced healthcare usage in both groups.

Multidisciplinary rehabilitation may offer the possibility of patient-centered care, with condition management being holistically adjusted to patients’ needs over time. A digital multimodal MSK program (as the herein reported) may address MSK pain through exercise and psychoeducation helping participants to change their beliefs about the causes and threat value of pain [99]. This may help them experience less fear of movement and more self-efficacy about their condition, thus reducing both anxiety and depressive symptoms exacerbated by MSK pain [100]. The exact weight that each DCP component has on the overall outcome change cannot be clarified in the current study design and should be further pursued in future trials.

### 4.3. Strenghts and Limitations

The major limitation of this study is the lack of randomization between groups, which impacts the establishment of causality of the intervention on the outcomes. Stronger evidence may be retrieved from future research using a randomized clinical trial considering conventional in-person therapy standard care as a control group. Nevertheless, the included cohorts have similar demographic patterns to the general US population [101], fostering generalization, and baseline differences were adjusted using inverse probability weighting and imputation for missing data to decrease bias. Additionally, despite weighting for levels of anxiety and depression at baseline, we cannot rule out the possibility that other psychological and cognitive traits (including motivation to perform the program) may have influenced the acceptance of the program and corresponding outcomes. Other limitations may include that the study design does not allow determination of the weight of each component of the DCP on the observed changes. In addition, while some people reported to have sought additional conservative and invasive and emergency care throughout the year, this was self-reported data (versus claims-based data). Additionally, we did not collect data about pharmacological usage, or other types of additional care, and therefore the influence of these on the clinical outcomes was not controlled. Nevertheless, the present study contributes to minimizing the gap in the literature regarding the long-term effect of a completely remote multimodal digital intervention.

## 5. Conclusions

This study was aimed at gaining further insight into the impact of a completely remote multimodal DCP in a real-world setting on long-term clinical outcomes of CMP. Participants going through the program reported sustained benefit both in pain and functionality at 1-year follow-up, as well as significantly higher improvements in pain and function in comparison to a control group of non-starters. This study suggests that a multimodal digital MSK program may have a lasting impact on improved pain and functionality in patients with chronic MSK pain.

## Figures and Tables

**Table 1:** Baseline characteristics of study sample and considering intervention and comparison group. Statistical analysis was performed between the intervention and comparison group.

		Group		
	Total (*n* = 867)	Intervention (*n* = 310)	Comparison (*n* = 150)	*p* Value
**Age (years), mean (SD)**	49.2 (11.4)	50.5 (11.3)	46.5 (11.1)	<0.001
**Age categories (years), N (%):**				
<25	9 (1.0)	3 (1.0)	2 (1.0)	
25–40	227 (26.2)	70 (22.6)	44 (29.3)	0.010
40–60	480 (55.4)	165 (53.2)	87 (58.0)	
>60	151 (17.4)	72 (23.2)	16 (10.7)	
**BMI, mean (SD)**	30.2 (7.4)	29.1 (6.6)	32.4 (8.5)	<0.001
**BMI categories, N (%):**				
Underweight (<18.5)	6 (0.7)	4 (1.3)	0 (0)	
Normal (18.5–25)	210 (24.2)	92 (29.7)	23 (15.3)	
Overweight (25–30)	291 (33.6)	97 (31.3)	48 (32.0)	<0.001
Obese(30–40)	274 (31.6)	93 (30.0)	48 (32)	
Obese grade III (>40)	86 (9.9)	24 (7.7)	31 (20.7)	
**Gender, N (%):**				
Woman	463 (53.4)	167 (53.9)	82 (54.7)	0.779
Man	402 (46.4)	142 (45.8)	68 (45.3)	
Nonbinary	2 (0.2)	1 (0.3)	0 (0)	
**Employment Status, N (%) ^a^:**				
Employed full time	518 (59.7)	157 (51)	29 (19.3)	
Employed part time	317 (36.6)	135 (43.5)	117 (78)	<0.001
Not employed	27 (3.1)	18 (5.8)	4 (2.7)	
**Anatomical Pain region, N (%):**				
Ankle	24 (2.8)	12 (3.9)	4 (2.7)	0.641
Elbow	22 (2.5)	6 (1.9)	5 (3.3)	
Hip	113 (13.0)	47 (15.2)	17 (11.3)	
Knee	135 (15.6)	41 (13.2)	24 (16.0)	
Low back	317 (36.6)	106 (34.2)	54 (36.0)	
Neck	98 (11.3)	33 (10.6)	20 (13.3)	
Shoulder	139 (16.0)	55 (17.7)	24 (16.0)	
Wrist/hand	19 (2.2)	10 (3.2)	2 (1.3)	

^a^ 5 missing values; BMI denotes Body Mass Index.

**Table 2:** Pain change analysis applying inverse probability weighting (IPW), considering either the entire cohort or just completers.

Variable	Estimate	Std. Error	df	t Value	*p* Value
**IPW + Completers**					
Comparison Baseline	4.79	0.17	821.63	28.89	
Comparison Post Change	−1.79	0.18	546.37	−9.76	<0.001
Intervention Baseline	4.57	0.20	819.36	−1.09	
Intervention Change	−2.38	0.22	543.47	−2.66	<0.001
**IPW + Entire cohort**					
Comparison Baseline	4.84	0.10	1550.14	49.18	
Comparison Post Change	−1.78	0.11	975.79	−16.05	<0.001
Intervention Baseline	4.62	0.14	1545.19	−1.56	
Intervention Change	−2.43	0.16	972.51	−4.10	0.008

**Table 3:** Odds ratio (OR) for being a responder for pain and functionality for the intervention group, and applying inverse probability weighting (IPW), considering both completers and entire cohorts.

Outcome	Model	OR	95% CI	z	*p* Value
Pain	IPW + Completers	1.90	1.27–2.86	3.09	0.002
	IPW + Entire Cohort	1.86	1.40–2.47	4.31	<0.001
Functionality	IPW + Completers	2.02	1.31–3.12	3.18	0.001
	IPW + Entire Cohort	2.25	1.72–2.96	5.85	<0.001

## Data Availability

The data presented in this study are available on request from the corresponding author. The data are not publicly available due to privacy restrictions.

## Supplementary Materials

The following supporting information can be downloaded at: https://www.mdpi.com/article/10.3390/healthcare10122349/s1, Table S1: Association of baseline variables with odds of being a responder for pain and disability.
