# Supplementary material for: Long-Term Clinical Outcomes of a Remote Digital Musculoskeletal Program: An Ad Hoc Analysis from a Longitudinal Study with a Non-Participant Comparison Group
Source: Healthcare (Basel). 2022 Nov 23;10(12):2349. doi: 10.3390/healthcare10122349 (PMC9778537; doi:10.3390/healthcare10122349)
Supplement: Supplementary file 1 [file healthcare-10-02349-s001.zip › healthcare-2000835-supplementary.pdf]

Supplementary Materials

Long-term clinical outcomes of a remote digital musculoskeletal program: an ad hoc analysis from a longitudinal study with a non-participant comparison group

Anabela C. Areias, Fabíola Costa, Dora Janela, Maria Molinos, Robert G. Moulder, Jorge Lains, Justin K. Scheer, Virgílio Bento, Vijay Yanamadala, Fernando Dias Correia

Supplementary Table S1 - Effect of each covariate in the odds for being a responder for pain and functionality.

Outcome	Model	Parameter	Estimate	SE	OR	95%CI		z	p
Pain	IPW + Entire cohort	(Intercept)	0.39	0.66	1.48	0.41	5.35	0.59	0.554
		Intervention	0.62	0.21	1.85	1.22	2.81	2.90	0.004
		Age	0.00	0.01	1.00	0.98	1.02	0.29	0.774
		BMI	-0.02	0.01	0.98	0.95	1.01	-1.37	0.170
		Gender=men	0.27	0.21	1.31	0.87	1.97	1.31	0.190
		Pain	0.12	0.06	1.12	1.00	1.26	2.01	0.045
		PHQ-9	-0.01	0.03	0.99	0.93	1.06	-0.24	0.809
		GAD-7	-0.06	0.03	0.95	0.89	1.01	-1.76	0.079
		Invasive and emergency care	-0.43	0.39	0.65	0.30	1.39	-1.12	0.264
		Conservative treatment	-0.22	0.27	0.80	0.47	1.36	-0.81	0.418
	IPW + Completers	(Intercept)	0.39	0.66	1.48	0.41	5.35	0.59	0.554
		Intervention	0.62	0.21	1.85	1.22	2.81	2.90	0.004
		Age	0.00	0.01	1.00	0.98	1.02	0.29	0.774
		BMI	-0.02	0.01	0.98	0.95	1.01	-1.37	0.170
		Gender=men	0.27	0.21	1.31	0.87	1.97	1.31	0.190
		Pain	0.12	0.06	1.12	1.00	1.26	2.01	0.045
		PHQ-9	-0.01	0.03	0.99	0.93	1.06	-0.24	0.809
		GAD-7	-0.06	0.03	0.95	0.89	1.01	-1.76	0.079
		Invasive and emergency care	-0.43	0.39	0.65	0.30	1.39	-1.12	0.264
		Conservative treatment	-0.22	0.27	0.80	0.47	1.36	-0.81	0.418
Functionality	IPW + Entire cohort	(Intercept)	0.67	0.63	1.96	0.57	6.71	1.07	0.286
		Intervention	0.65	0.21	1.92	1.28	2.89	3.13	0.002
		Age	0.00	0.01	1.00	0.98	1.01	-0.47	0.636
		BMI	-0.03	0.01	0.97	0.95	1.00	-2.09	0.037
		Gender=men	-0.23	0.20	0.80	0.54	1.17	-1.16	0.248
		Pain	0.07	0.05	1.07	0.96	1.19	1.29	0.196
		PHQ-9	0.03	0.03	1.03	0.96	1.10	0.78	0.433
		GAD-7	-0.06	0.03	0.94	0.88	1.00	-1.85	0.064

	Invasive and emergency care	-1.02	0.41	0.36	0.16	0.80	-2.49	0.013
	Conservative treatment	0.34	0.27	1.40	0.83	2.37	1.27	0.203
IPW + Completers	(Intercept)	0.47	0.69	1.60	0.41	6.20	0.68	0.499
	Intervention	0.72	0.22	2.05	1.32	3.18	3.19	0.001
	Age	0.00	0.01	1.00	0.98	1.01	-0.47	0.640
	BMI	-0.02	0.02	0.98	0.95	1.01	-1.16	0.244
	Gender=men	-0.16	0.21	0.85	0.56	1.29	-0.77	0.439
	Pain	0.04	0.06	1.04	0.93	1.17	0.71	0.480
	PHQ-9	0.03	0.04	1.04	0.96	1.12	0.90	0.368
	GAD-7	-0.06	0.04	0.94	0.87	1.01	-1.78	0.076
	Invasive and emergency care	-0.86	0.46	0.42	0.17	1.04	-1.88	0.060
	Conservative treatment	0.28	0.28	1.33	0.76	2.309	1.002	0.316
